# Advancements in Drug Delivery Systems for the Treatment of Sarcopenia: An Updated Overview

**DOI:** 10.3390/ijms251910766

**Published:** 2024-10-07

**Authors:** Alfred Najm, Elena-Theodora Moldoveanu, Adelina-Gabriela Niculescu, Alexandru Mihai Grumezescu, Mircea Beuran, Bogdan Severus Gaspar

**Affiliations:** 1 Department of Surgery, Carol Davila University of Medicine and Pharmacy, 050474 Bucharest, Romania; alfred.najm@yahoo.ro (A.N.); drmirceabeuran@yahoo.com (M.B.); bogdan.gaspar@umfcd.ro (B.S.G.); 2 Emergency Hospital Floreasca Bucharest, 014461 Bucharest, Romania; 3 Department of Science and Engineering of Oxide Materials and Nanomaterials, National University of Science and Technology Politehnica Bucharest, 011061 Bucharest, Romania; moldoveanu.theodora99@gmail.com (E.-T.M.); adelina.niculescu@upb.ro (A.-G.N.); 4 Romania Research Institute of the University of Bucharest—ICUB, University of Bucharest, 050657 Bucharest, Romania

**Keywords:** sarcopenia, drug delivery, treatment, muscles, aging of muscle

## Abstract

Since sarcopenia is a progressive condition that leads to decreased muscle mass and function, especially in elderly people, it is a public health problem that requires attention from researchers. This review aims to highlight drug delivery systems that have a high and efficient therapeutic potential for sarcopenia. Current as well as future research needs to consider the barriers encountered in the realization of delivery systems, such as the route of administration, the interaction of the systems with the aggressive environment of the human body, the efficient delivery and loading of the systems with therapeutic agents, and the targeted delivery of therapeutic agents into the muscle tissue without creating undesirable adverse effects. Thus, this paper sets the framework of existing drug delivery possibilities for the treatment of sarcopenia, serving as an inception point for future interdisciplinary studies.

## 1. Introduction

Muscles are an important component of the human locomotor system, which helps in the locomotion process and plays other important roles in the body. Muscles are involved in metabolic processes, especially glucose metabolism, directly or indirectly influence bone density, store amino acids essential for protein synthesis, and produce myokines. The occurrence of a muscle disorder can lead to major imbalances in the human body [1].

Sarcopenia is a progressive geriatric syndrome, which is characterized by loss and aging of muscle mass but also of muscle properties, with a relatively high incidence in older people. However, sarcopenia can also occur in mid-life and has been associated in some patients with other diseases such as cancer, liver dysfunction, renal dysfunction, or metabolic disorders. Studies have concluded that this condition involves several processes that lead to loss of muscle function through denervation, inflammation, hormonal dysregulation, and mitochondrial dysfunction, which implicitly alter patients’ quality of life but may also lead to increased mortality. In principle, aging is one of the main causes of sarcopenia, but lack of proper nutrition, sedentary lifestyle, obesity associated with its complications, sudden and uncontrolled weight loss, diabetes, chronic inflammation, hormonal imbalances, and genetic factors are associated with an increased risk of developing this condition [2,3,4,5]. Hormonal dysregulations play an important role in the onset and progression of sarcopenia. For example, decreased insulin sensitivity and the development of type 2 diabetes are closely linked to this condition, due to the function of muscles to ensure energy homeostasis by regulating metabolism and insulin sensitivity. In this respect, skeletal muscles contribute to the synthesis of myokines and muscle cytokines that have anti-inflammatory effects on excess visceral fat and improve insulin action. Loss of muscle mass may decrease insulin secretion and affect glucose transport into muscle cells via GLUT3 receptors, and increased lipid accumulation in muscle contributes to mitochondrial dysfunction, conducing to insulin resistance and decreased glucose metabolism in muscle. Another hormone with a major impact on sarcopenia is cortisol, which is secreted by the adrenal glands and is known as the stress hormone. It plays a major role in the breakdown of muscle protein. Aging increases the secretion of this hormone, which can reduce muscle mass due to muscle proteolysis. Another hormone secreted by the adrenal glands is dehydroepiandrosterone (DHEA), which decreases with age. Studies show that DHEA increases the levels of growth factors (e.g., IGF-I) and testosterone, which help maintain muscle mass, and that it can affect muscle mass by decreasing. Testosterone and estrogen are two important hormones for maintaining muscle mass. Decreased production of these hormones has been associated with a considerable decrease in muscle mass and strength. Testosterone promotes protein synthesis and muscle growth, leads to proper proliferation of satellite cells, and actively contributes to muscle regeneration, while estrogen has effects on the contractile properties of muscles and contributes to muscle regeneration. Adipokines are secreted by adipose tissue and have effects on metabolism and inflammation. The more they are secreted, the greater the risk of developing sarcopenia and obesity. Also, the hormone responsible for regulating blood pressure, angiotensin II, has effects on muscles. Increased levels of angiotensin II may lead to decreased muscle strength and may activate muscle proteolysis. Thus, IGF-I signaling pathways decrease, and the risk of apoptosis increases, contributing to muscle atrophy [6,7,8].

At the same time, as muscles age, mitochondria, which are responsible for producing cellular energy, begin to show several dysfunctions, starting with the generation of reactive oxygen species (ROS), which contribute to progressive muscle cell damage with increasing age. Aging can also affect the processes that synthesize new mitochondria, such as mitophagy and biogenesis, which can be maintained with exercise. Also, by accumulating excess ROS, mitochondria can promote apoptosis by opening the mitochondrial permeability transition pore (mPTP), leading to the release of cytochrome c and activation of proapoptotic processes. Thus, in elderly people, mitochondrial function deteriorates, resulting in sarcopenia. It has been observed that mitochondrial mass is considerably reduced, and the activity of respiratory enzymes and ATP-producing capacity is affected, leading to reduced muscle homeostasis and muscle strength [9,10,11]. Thus, both hormonal imbalances and mitochondrial dysfunction are major factors in the onset and evolution of sarcopenia and may represent a starting point for an optimal treatment against this condition.

Sarcopenia can affect a patient’s lifestyle by restricting movement, leading to obesity and osteoporosis, producing a metabolic and hormonal imbalance and, therefore, a decrease in quality of life [12]. There is an association between cachexia and sarcopenia, particularly in elderly cancer patients. Cachexia is also a term attributed to significant loss of muscle mass, often accompanied by systemic diseases such as cancer, end-stage renal disease, or cardiomyopathy. It is also considered a complex metabolic syndrome characterized by loss of muscle, either by loss of mass or fat loss, so that patients lose their physical functionality. Cachexia could be related to symptoms such as inflammation, insulin resistance, breakdown of muscle proteins, and anorexia, all of which are points of identification and association with sarcopenia. Also, oncologic research attributes the term sarcopenia to the body composition of patients, referring to a considerable reduction in muscle mass. Thus, some patients suffering from cachexia may also develop sarcopenia, reporting not only loss of muscle mass but also loss of muscle function. In this respect, there is no well-established protocol regarding treatment methods for sarcopenia or cachexia. Lifestyle changes, physical training, and supplementation could lead to symptom relief and reduce the chances of both conditions worsening, but not considerably. Also, as the two are associated, possible strategies to develop innovative treatments could be applied to cachexia [13,14].

However, the causes of sarcopenia are not fully understood. Research needs to continue to define the complex relationship between chronic inflammation, hormonal dysregulation, mitochondrial dysfunction, and loss of muscle mass and function with age. At present, the only treatment strategies for sarcopenia are lifestyle changes, supplementation, and endurance exercise, but these do not have a significant effect on improving or treating sarcopenia, and studies need to continue to develop an effective and safe treatment for this condition [2,3,4,5,15].

One of the problems with this syndrome is the lack of standard methods for early diagnosis of this disease and the long time for diagnosis with current methods. At present, SARC-F, together with measurement of the calf circumference, is the main early diagnostic method for sarcopenia, which tests muscle function. Following the SARC-F test (Strength, Gait Assistance, Chair Rise, Stair Climbing, and Falling), a total score of ≥4 points may indicate an increased risk of developing sarcopenia. However, ultrasound (US), computed tomography (CT), magnetic resonance imaging (MRI), dual-energy X-ray absorptiometry (DXA), and bioelectrical impedance assessment (BIA) are methods that can assess the stage of muscle weakness. Muscle strength can also be assessed by knee flexion and extension, handgrip strength, and maximal respiratory output, while stand-up tests, walk tests, long-distance walking tests and body mass index (BMI) are increasingly used to assess muscle function. Late diagnosis of sarcopenia, when a considerable loss of muscle mass has set in, is another major problem. A challenge may be to improve early diagnostic strategies using advanced techniques such as those mentioned above [16,17].

In the search for ways to relieve the symptoms of sarcopenia, many clinical studies have focused on developing possible treatments. Thus, physical exercises are recommended, especially endurance and aerobic training, which have been shown to improve muscle strength and performance. At the same time, they have a role in decreasing inflammation, inducing angiogenesis, and promoting the secretion of growth factors, both muscle and bone. Although an effective method, exercise cannot be used for patients who are already immobilized or have limited movement. The administration of nutraceuticals (supplements of amino acids, vitamins, or small compounds) is also considered an alternative, even if no noticeable effects on the evolution of patients have been observed [17,18].

At present, research is continuing to find an alternative to help manage sarcopenia, with efficient drug delivery being a key starting point for studies. Thus, the development of targeted delivery systems to the muscle using specific proteins on the surface of muscle cells is being pursued to minimize the side effects of systemic administration. The use of nanoparticles is also an approach in the treatment of sarcopenia, as they can support myoblast proliferation and, thus, muscle regeneration. Another variant would be to obtain scaffolds that mimic the extracellular matrix of muscle cells so that myogenesis is favored. Peptide delivery is another option that is effective in treating sarcopenia. Adeno-associated viral vectors are mainly used in muscle disorders (e.g., Duchenne muscular dystrophy, spinal muscular atrophy) but have also been studied for the treatment of sarcopenia but have limitations such as a low capacity to edit nucleic acid for the regeneration of aged muscles. Natural systems, such as extracellular vesicles, are ideal for delivering functional proteins and nucleic acids, which can be used in muscle regeneration and metabolism. Another approach may be the exogenous administration of stem cells or progenitor cells [2,19].

However, the main issue remains the development of effective treatments and how they should be optimized for administration. Although there is currently no ideal treatment approved for sarcopenia, studies are continuing to develop curative delivery systems. In doing so, researchers need to consider the characteristics of muscle aging, as changes in satellite cells have the potential to reveal new therapeutic targets. Also, the construction of delivery systems must be developed with the mechanisms of muscle regeneration in mind to enable regeneration and limit muscle loss caused by aging [2,19].

## 2. Drug Delivery Systems and Formulations

### 2.1. Overview of Drug Delivery Approaches

A drug delivery system (DDS) might represent a device or formulation that transports therapeutic agents or pharmaceutical compounds to an intended part of the body. These systems can enhance the therapeutic agent’s efficacity and safety by controlling the release rate and time, protecting the active ingredients from the body’s aggressive environment. A drug delivery system can be considered a successful alternative when the therapeutic effect is maximized and the accumulation of a drug concentration at the administration site is minimized. Traditional methods of drug administration have, during their use, presented numerous problems that have diminished the therapeutic effect of the drugs. They were either delivered in a high dose, with low availability, or were unstable and released the active substance too quickly. Thus, drug delivery systems aim to mitigate and solve these drawbacks, offering the possibility of conferring increased performance, protection, and comfort to patients [20,21,22,23,24].

Drug delivery systems can be classified into two major categories: traditional/conventional drug delivery systems and novel drug delivery systems/controlled-released drug delivery systems. Traditional drug delivery systems are classical drug delivery methods, with the main purpose of rapid delivery and absorption of the therapeutic agent. These DDSs are generally found in simple forms and can be administered orally, nasally, transdermally, or intravenously. In this way, they can be classified by the route of administration as oral, nasal, transdermal, parenteral, vaginal, and rectal DDSs. However, these delivery systems also have disadvantages, as they do not maintain their drug concentration constant over time, which implies multiple administration of the drug at regular intervals. To improve the therapeutic effect, novel drug delivery systems (NDDSs) have emerged, which can control drug release, proper dosing, much better action, and targeting of the desired tissue. Although NDDSs have numerous advantages, such as extending the duration of action and bioavailability of the drug, reducing the dose and decreasing the frequency of administration, protecting the active substance, and reducing adverse effects in local administration, they present an increased risk of toxicity, the need for interventions to apply or remove the NDDS, experiences of discomfort for patients (in the case of implants), and increased cost. NDDSs can also be classified into four subcategories: rate-preprogrammed, activation-modulated, feedback-regulated, and site-targeting delivery systems, which offer innovative and efficient means of drug delivery [25,26].

For a drug delivery system to be used for therapeutic purposes, several factors must be taken into account that lead to increased effectiveness of these systems. First, the properties of the drug must be considered, such as solubility, molecular mass, stability, and half-life, which are important aspects that may subsequently influence the way the system is administered. For example, a drug-based system with high solubility (e.g., a lipid-based DDS) can be administered orally. Another important aspect regarding a DDS may be the target site, which may also be influenced by the properties of the drug and the site where the drug is administrated. The route of administration and the release profile of the DDS may also be important and are influenced by the properties of the drug, the site of administration where the DDS actionizes, and minimally invasive interventions. Other aspects, such as the patient’s condition, safety, and ease of manufacture of the DDS, are crucial factors in achieving functional systems [26,27].

Drug release kinetics is based on two important concentrations: the minimum effective concentration and the toxic concentration, which are important in designing and developing a DDS. The minimum effective concentration is the concentration at which the drug has no effect, while the toxic concentration is the concentration at which adverse effects begin to appear. For the drug to have the desired therapeutic effect and to be safe to use, its concentration must be maintained between the two concentrations mentioned above. The therapeutic index is also a parameter indicating the relative safety of a drug. With the help of this parameter, it is possible to determine the therapeutic effect of the drug and the amount at which it becomes toxic [21].

**Figure 1:**
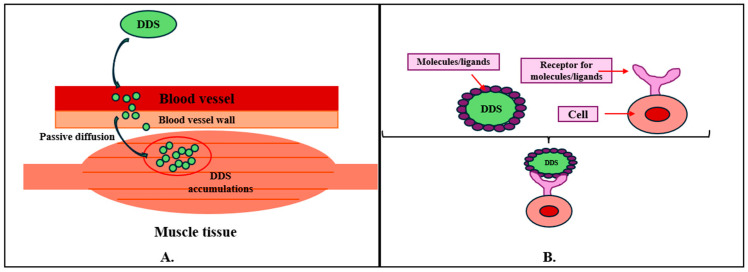
Illustration of targeted drug delivery mechanisms. (**A**) Passive targeting; (**B**) active targeting.

The mechanisms of drug delivery have been intensively studied in the context of sarcopenia to deliver drugs to the muscles better, to minimize as much as possible the adverse effects, and to increase the therapeutic effect. In this regard, drug targeting is categorized into two methods: passive targeting (Figure 1A) and active targeting (Figure 1B). In passive targeting, due to the physicochemical characteristics of the drugs, they accumulate in tissues such as muscles. This type of delivery relies on the properties of blood vessels, such as increased permeability, which allows DDSs to penetrate tissues. On the other hand, active targeting involves molecules or ligands that bind directly to cell receptors. For example, to develop a specific DDS to treat sarcopenia, nanoparticles have been created that target mitochondria receptors in muscle to improve mitochondrial function and its effects on muscle tissue aging [28].

### 2.2. Importance for Sarcopenia Treatment

In recent years, the development of therapeutic strategies against sarcopenia has focused on the use of drugs commonly prescribed for other conditions (e.g., testosterone, levothyroxine, melatonin, oxytocin), but also on sustained exercise. As the incidence of this disease has increased, with 1.71 billion people suffering from diseases of the musculoskeletal system, regenerative drug therapies are beginning to be studied in depth. However, significant barriers need to be overcome, such as the difficulty of effective drug delivery into tissues. In addition, studies must focus on the development of DDSs that take into account the route of administration, biomolecule interactions, interaction with body fluids, and other issues related to the loading of delivery systems and precise tissue targeting. Also, the disease stage can greatly influence the action of DDSs at the tissue level and should be taken into account when developing DDSs. Targeting fragments should be added to the drug or delivery system to improve the delivery method of the DDS. The DDS should also possess an optimal size and be properly loaded to penetrate the matrix and target the tissue [29].

## 3. Oral Delivery Systems

### 3.1. Advantages and Challenges

The oral route of administration is one of the most widely used in systemic and local drug delivery due to the fact that it is easy to use for patients, has the feasibility of solid formulation, is low cost, and is non-invasive. However, this delivery method remains a challenge as oral delivery systems have to cope with the aggressive environment of the gastrointestinal tract. In addition, an ideal DDS should allow a drug to be delivered to a target tissue in an appropriate concentration but also with an easily modifiable dosage [30,31].

In reality, these systems have poor bioavailability and limited targeting because of the gastrointestinal tract and hepatic metabolism. Also, some patients have nutritional problems because of muscle mass and function loss, which affect their medication intake. Comorbidities may also affect the sarcopenia drug intake because of possible interaction with other medications. So, for the treatment of musculoskeletal disorders such as sarcopenia, local administration of drugs is preferred since a controlled release of an optimal concentration of the drug is desired over a long period. However, if the barriers imposed by the gastrointestinal tract were overcome, the use of orally administered DDSs would remain an easy and patient-friendly medication option for patients who are suitable for oral administration [15,32,33,34].

Thus, to develop DDSs for this purpose, one has to take into account drug proproteins, physiological factors, and the anatomy of the digestive system, which play an important role in both digestion and drug absorption. Lipinski’s rule of five (ROF) is used in the development of new drugs with superior therapeutic effects, defining the drug’s availability. The molecular mass should not exceed 500 Da, logP (the log of the partition coefficient of a molecule between two phases) values should not be less than 5, the donor hydrogen bonds should be 5, and the acceptor hydrogen bond number should not be more than 10. Thus, the ROF score should not be between zero and four. In this regard, conventional delivery systems (tablets or capsules) have a low therapeutic effect due to low drug accumulation at the specific site, inadequate distribution in the body, and the occurrence of unrecognized side effects. Due to the need for the development of improved DDSs to reduce these drawbacks, nanoparticles have started to be studied to improve the biodegradability of drugs, to provide for more precise drug targeting, and to make them more specific in terms of pharmacology [30,31,35].

### 3.2. Oral Drug Delivery System

**Figure 2:**
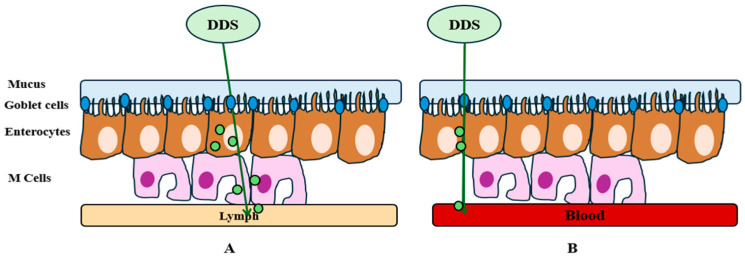
Schematic of a drug delivery system’s action through the mucus layer. (**A**) Transcellular transport; (**B**) paracellular transport. Created based on information from [36,37,38].

As for the challenges of oral delivery systems, these are found in the gastrointestinal tract, where absorption takes place and enters the bloodstream to act in different body parts. Thus, drug absorption occurs in the small intestine, where the pH, presence of enzymes, and residence time can affect the efficient absorption and integrity of the therapeutic agent. Biological barriers are represented by pH and enzymes in the stomach and intestine, which denature and degrade drugs, while mucus, a protective lining of the gastrointestinal tract, can prevent efficient absorption (Figure 1) [36]. Enterocytes are the first line of cells present in the small intestine that are responsible for the absorption of nutrients and drugs from the intestinal epithelium. These cells are responsible for the absorption of lipophilic substances, and absorption occurs by passive diffusion through the membrane of the enterocytes. M cells are also involved in the transport of DDS or macromolecules into the lymphatic system, protecting them from first-pass metabolism. Goblet cells are responsible for mucus secretion and can act both as a protective barrier and as a facilitator for drug absorption. Thus, some DDSs are designed to penetrate or interact with the intestinal mucosa and improve bioavailability [37,38]. Tissue barriers prevent drug absorption, and this depends on the size and chemical properties of the drugs. In general, large, hydrophobic molecules tend to be absorbed transcellularly (Figure 2A), while small, hydrophilic molecules are absorbed paracellularly (Figure 2B) [36].

Paracellular transport is realized by passive diffusion due to the presence of tight junctions present between epithelial cells, thus allowing the passage of molecules with diameter sizes between 22–30 Å, while larger molecules such as proteins (>100–200 Da) are transported transcellularly. Transcellular transport is achieved either by passive or active diffusion using specific transporters for each type of cargo (e.g., amino acids, fatty acids, sugars). Thus, receptors and passive transporters are involved in the transport of orally administered drugs, and among the best known are ATP-binding cassette (ABC) transporters, such as P-glycoprotein (P-gp), which can limit the absorption of some drugs and contribute to their elimination from epithelial cells in the intestinal lumen, and multidrug resistance-associated cassette (MRP) transporters, which control the permeability of drugs in the small intestine. Also involved in active transport are the bile receptors ASBT (apical sodium bile acid transporter) and other nutrient transporters such as PEPT1 (peptide transporter 1). In this regard, lipid nanoparticles and peptide-penetrating systems are being studied to more efficiently transport orally administered active substances [39,40].

For the treatment of sarcopenia, oral medications are not an option. Because sarcopenia is a chronic condition that requires constant treatment, this route of administration may be ineffective, as patients may find it difficult to take the drugs consistently due to comorbidities or the progression of sarcopenia. Also, the disadvantages of this route of administration are another reason why it may be ineffective and may not lead to an improvement in a patient’s health [29,41].

In this way, oral drug delivery systems need to possess advanced properties to overcome challenges such as sustained delivery, solvent-free microencapsulation techniques, co-delivery systems, and the mass production capacity of these systems [36].

### 3.3. Recent Advantages in Oral Formulations

A strategy to improve oral formulations could be to improve the delivery methods of drugs with low water solubility, which presents a reduced bioavailability. Thus, it is proposed that surfactants be used to facilitate drug absorption via the presence of hydrophilic components as well as a hydrophobic component, and the drug should be placed at the interface between the two. However, this approach may present safety issues. Also, in terms of the use of micro/nano particles whose size is easily controllable, small particles have a large specific surface area, which may lead to higher drug absorption [42,43].

Also, the oral absorption of drugs can be improved by various drug modifications. Salt formation can increase the absorption of drugs with a weak acidic or basic character by changing the pH during drug dissolution. However, this method takes time and involves much trial and error until a favorable result is obtained. The chemical modification and emergence of prodrugs have led to the emergence of chemical derivatives of the main drugs, so they require enzymatic transformation to have a therapeutic effect. Prodrugs need to be inert and non-toxic and easily metabolized. They have been shown to increase oral bioavailability by increasing drug solubility and intestinal permeability [42].

Other methods involve the complexation of drugs and the formation of inclusion complexes with drug molecules, which increases the solubility of the drug and its release rate. Cyclodextrins are complexing agents that form inclusion complexes with non-polar molecules, increase the stability of drugs in aqueous media, and make them more soluble [42,43].

Lipid-based formulations have been developed to increase the dissolution rate and solubility of a drug in the gastrointestinal tract. They also have the advantage of avoiding the first hepatic passage, and these formulations may inhibit efflux pumps [42].

Micellar carriers or polymeric nanocarriers are also used to improve the bioavailability of drugs and efficient delivery. These have the role of increasing solubility, especially for lipophilic or water-insoluble formulations for oral administration. Micellar polymers are formed in surfactant solutions and are used to incorporate lipophilic drugs. Such a purifier consists of hydrophilic and hydrophobic polymer blocks with a lipophilic core, which is the container of the drug to be delivered. Also, these carriers are sensitive to pH changes but can be chemically modified to deliver targeted therapies, thereby increasing bioavailability and enhancing disease-specific therapeutic effects compared to regular suspensions [42]. pH-sensitive micelles may respond to endogenous factors such as low interstitial pH or interact with enzymes that may be present in high concentrations. Thus, at the cellular level, micelles can release a drug depending on the pH of the environment, creating the possibility of controlled-release DDSs for sarcopenia [32].

Nanocarriers have demonstrated an increased ability to deliver therapeutic agents and may provide an efficient method of controlled drug delivery with the potential to increase the therapeutic agent’s specificity, efficacy, and tolerability [42,44].

In order to achieve sustained delivery, which consists in maintaining an optimal concentration of the drug in the blood without fluctuations that produce side effects, micro adhesive carriers have been developed. These carriers have electrostatic properties that help them form bonds with mucus, but they are not effective over time due to the renewal of the mucus layer in the intestine [44]. Another strategy would be O-rings, which can extend the time, but they are not safe for use and still need to be studied [42].

Solvent-free microencapsulation is a technique that has been developed to limit the contact of organic solvents during the production of microspheres (solid drug dispersed in a polymeric matrix or empty drug encapsulated in a polymeric shell), which could distort the drug. Water-in-oil and oil-in-water techniques are used to reduce solvent contact but are complex and have stability issues [42].

Co-delivery systems are designed to deliver effective treatments simultaneously to different sites and reduce drug adverse effects and ineffectiveness. To develop such a system, one approach has been to encapsulate drugs in separate vehicles (micelles, liposomes, or microparticles), and to use coatings to enable controlled drug release. This technique allows both controlled drug release and specific delivery with a much-improved therapeutic effect [36].

Although these methods are promising and may help overcome the limitations associated with oral administration, some challenges need to be overcome before they can be utilized in the long term. Adverse effects and long-term stability of these DDSs should be a long-term research goal to come up with an improved version that will allow the commercialization of such DDSs [42].

Currently, studies are underway to develop treatments administered through oral delivery systems, including enzyme inhibitors, receptor agonists, and drug repurposing. First, one study [45] focused on the repurposing of niclosamide, which has been shown to inhibit signaling for pathways like STAT3 and Wnt/β-catenin associated with muscle atrophy. Studies in vitro and in experimental models have demonstrated the potential to inhibit muscle atrophy and cachexia, and it may also be used in the treatment of sarcopenia. Another example [46] may be ghrelin agonists (e.g., anamorelin), which have been shown to improve muscle growth and muscle strength by activating ghrelin receptors. Anamorelin also stood out due to its high bioavailability following oral administration, making it a candidate for the possible treatment of sarcopenia. Another study demonstrated that β2-adrenoceptor agonists (e.g., formoterol and clenbuterol) may slow muscle wasting by activating signaling pathways involved in protein synthesis, a potential candidate in treating sarcopenia. However, there is a risk of cardiovascular complications [46].

## 4. Injectable Delivery Systems

### 4.1. Intramuscular and Subcutaneous Injections

Injections have become one of the most popular methods of administering medicines because of their advantages. Since oral administration of medicines has the disadvantage of physiological barriers, and some medicines cannot be processed for oral administration, injections have proved to be more practical. Injections have the advantage of delivering a drug more quickly, directly, and accurately, and this process can be accomplished by three routes: intramuscular (IM), subcutaneous (SC), and intravenous (IV) [47,48].

Routes of injectable drug administration may depend on drug-related principles, such as safety and efficacy, or factors related to patient preferences, health status, and pharmacoeconomics. Table 1 highlights factors that may drive the choice of route of administration [47,48].

IM injections are preferable when administering drugs such as anti-emetics, hormones, analgesics, and sedatives, but also in terms of immunization because striated muscles are richly vascularized and the drug enters the systemic circulation rapidly. In order to treat sarcopenia, the injection of insulin, growth hormones (hGH and IGF-1), testosterone, and epinephrine led to improved protein synthesis in muscle. In addition, peptides and dendrimers have been tested to obtain a therapeutic vaccine for muscle regeneration, together with cell injection and myostatin inhibitors that are being targeted for such applications [49,50,51,52].

Water-soluble drugs (e.g., insulin, epinephrine) have the ability to be absorbed more rapidly, while drugs that are administered using an oil-based vehicle show slow diffusion [53,54]. This method has demonstrated a rapid drug uptake and a prolonged pharmacokinetic release, being easily dissolved and penetrating into the blood circulation, with sustained action and increased bioavailability [48,49,50,51,52].

Also, the occurrence of infections or nerve damage may occur following IM injections due to the wrong choice of administration site or incorrect administration [55,56,57].

SC injections are similar to IM injections and can be used to avoid drug inactivation or the limitations of oral administration. Many drugs can be administered by this method, which can retain properties such as bioactivity and rapid absorption. As with IM injections, pain is an unpleasant factor for patients, and this is due to the characteristics of the needle used or the injection site. The volume of drugs is also a factor that can cause pain, which is why volumes are reduced, but also the speed at which they are introduced. This method is preferred when there is a risk of muscle tissue damage with IM injections or when the release into the circulatory system is too slow [58,59].

IM injections are generally used because of their faster effect at maximum concentrations and in emergencies (e.g., epinephrine). However, they may present increased discomfort and local damage, in contrast to SC injections, which offer better comfort. This alternative may be slower in reaching optimal concentration and is preferable in long-term treatments [48,59].

### 4.2. Innovations in Injectable Formulations

Injectable forms are the use of medicines in critical situations. Recently, for the creation of injectable delivery systems, effective formulations, controlled drug release, biodegradability (e.g., polymers), and the elimination of discomfort (e.g., microbeads) have started to be studied [60]. These can improve accuracy and safety, reduce unrecognized adverse effects, and promote targeted delivery. Formulations aimed at release control tend to prolong the release time of drugs and reduce the frequency of injections, but also establish an optimal dosage. These formulations are generally a good option in the treatment of frame diseases. Biodegradable polymers have the advantage of releasing active substances as they break down into non-toxic elements in the body [61]. They promote sustained release of the drug and thus minimize the occurrence of side effects. Micro-needles are a minimally invasive alternative, which is generally relevant for vaccines or insulin administration in diabetic patients. The advantages that innovations can bring to these drug delivery systems are the prolongation of the therapeutic effect of the active substance and the decreased need for frequent dose administration. They can be used in the treatment of chronic diseases, making life easier for patients [62].

## 5. Transdermal Delivery Systems

Transdermal drug delivery is a painless and non-invasive systemic drug delivery method involving applying formulations directly to healthy skin. To do this, the drug must penetrate the leg’s stratum corneum and deep into the layers without accumulating in the dermis, where active substance absorption occurs. Transdermal delivery systems (TDDs) reduce adverse effects and can improve stability and enzyme degradation, as with oral administration, while avoiding first-pass metabolism. Thus, drugs are released sustainably, with optimal plasma concentration. Over the years, various methods of using TDD have been developed, including obtaining films, dressings, or gels, which have been designed into formulations to facilitate drug delivery. Table 2 shows the main advantages and disadvantages of TDDs [63,64,65,66].

**Figure 3:**
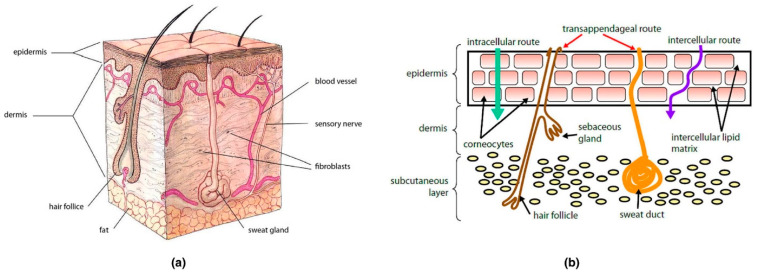
Skin layers (**a**) and molecule’s absorption through skin layers (**b**). Reprinted from an open-access source [66].

Although they have many advantages, the main challenge that TDDs present is the skin itself. The skin (Figure 3a) is the outer organ intended to protect the body from environmental factors (e.g., toxins, heat, chemicals). It is also a multilayered structure consisting of the epidermis (the outer layer) and the dermis (the layer containing the blood vessels). The outer stratum corneum of the skin (SC) prevents transdermal administration by having lipids and hydrophilic and hydrophobic substances on the skin, making the skin impermeable to most pharmaceuticals and small molecules. However, at the SC level, molecules can pass through via three modes, transcellular, intercellular, or transappendageal (Figure 3b), and then diffuse to reach the dermis [66,67].

### State-of-the-Art Technologies

To facilitate drug delivery by the transdermal route, techniques have emerged to improve TDDs. These techniques are categorized into active (iontophoresis, electroporation, sonophoresis, photomechanical waves, thermal ablation, and microneedle) and passive (polymeric nanoparticles) [67].

Iontophoresis is a continuous, fast, and easy delivery method that can improve the delivery of polar molecules as well as molecules with high molecular mass. However, it may present an increased risk of burns, the therapeutic agent is difficult to introduce and stabilize in the delivery vehicle, and the delivery is too complex [67,68,69]. A recent study conducted by Kohki Michiue et al. led to the development of an intradermal delivery system using iontophoresis to administer myostatin inhibitory-D-peptide-35 (MID-35) in a mouse model. This study demonstrated that MID-35 delivery led to an increase in muscle mass. Modifications in mRNA levels of downstream genes of myostatin were also observed, highlighting the potential use of the method in the treatment of sarcopenia [51].

Electroporation allows direct drug transfer but cannot be used over large areas. Delivery can be interrupted due to a voltage that is too high, and tissue can be easily injured. It also presents an increased risk of infection [67]. A study by Anna Stephan et al. used electroporation to deliver siRNA into the tibialis anterior muscles in a mouse model, with the aim of temporarily reducing messenger RNA levels and manipulating gene functions in the muscle. The effect of electroporation on mRNA is transient, being significant for a short period of 2–3 weeks, without affecting long-lived proteins [70].

Sonophoresis is an appreciated and common technique in many patients, which allows control of the diffusion of transdermal drugs and leads to the removal of blood clots. However, the administration time is prolonged, with the risk of skin lesions, and the SC must not already be lesioned for the administration to be effective [67]. This technique is used for hydrophilic drug delivery, macromolecule transportation, and in gene therapy and nanocarrier delivery. This technique has also proven to be useful in treating injuries to tendons, ligaments, and cartilage, as well as in drug delivery for skeletal muscle disorders such as sarcopenia [71].

Photomechanical waves have been shown to produce no skin lesions, discomfort, or pain, but more studies are required for safe use in patients [67]. This technique may be of use for sarcopenia treatment given that it has been used in experimental muscle tissue restoration, both alone and in combination with other methods [72].

Microneedles are painless and allow for a treatment to be applied to a specific area of the skin and for rapid healing at the injection site. However, a low dose can be administered compared to hypodermic needles, and the depth to which the microbeads penetrate may depend on the thickness of the skin layers. Tissue damage may also occur due to repeated injections [67]. It has been shown that microbeads have considerably reduced the adverse effects associated with traditional drug administration in skeletal muscle-related diseases, with a high potential for sarcopenia treatment [73].

Polymeric nanoparticles can enable targeted and controlled drug delivery by being tough and non-deformable. An important advantage of these is using biodegradable polymers, which allow the loading of hydrophobic and hydrophilic drugs [38]. In one study, polymer nanoparticles were used to create a drug delivery system in the muscle. This study is relevant for sarcopenia as it links to functionalization with muscle-homing peptides that showed a significant increase in cellular uptake of the nanoparticles, with improved therapeutic effects and decreased risks of adverse effects [74].

In order to improve such systems, direct and passive methods offer opportunities for their use in the treatment of various diseases. Active/physical techniques have limitations that do not allow the use of large doses of hydrophobic drugs, and their stability is poor, uncontrollably releasing the therapeutic agent. In this sense, the use of nanomedicine, together with these methods, may lead to the emergence of new therapeutic opportunities, aiming to improve the solubility, stability, and bioavailability of drugs. For example, microbeads and nanoparticles have long-lasting effects, while hybrid microbeads–solid dispersion systems improve transdermal penetration and efficient drug delivery [65].

Regarding the treatment of sarcopenia using transdermal routes, one of the most popular methods is the use of a testosterone-based gel. A few studies have shown that this method has been shown to improve muscle strength and mobility, but adverse effects such as cardiovascular problems, prostatic hyperplasia, allergic reactions, thrombosis, and ultimately prostate cancer have also been reported. Research is currently being carried out to minimize possible adverse effects by formulations that allow testosterone to be used with other drugs in order to be used safely [75,76,77,78].

Although an easy drug delivery method, TDDs are difficult to manufacture and present high costs in order to develop the final dosage forms. Also, another challenge is the delivery of large macromolecules (e.g., proteins) using nanomedicines. Therefore, there is still a need for further research to provide conclusive information about their therapeutic potential and to overcome the limitations that TDDs present, and their subsequent use commercially [65].

## 6. Nanotechnology-Based Drug Delivery Systems

**Figure 4:**
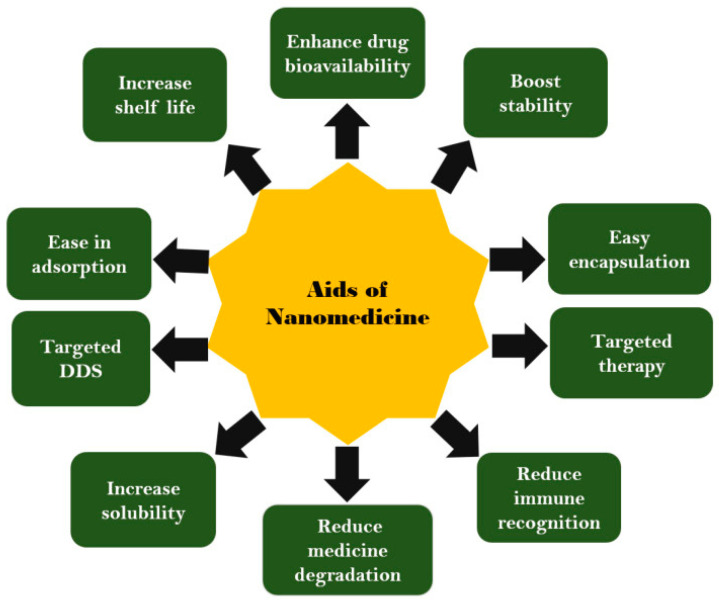
Main purposes of nanomedicine. Reprinted from an open-access source [83].

Nanotechnology is a branch of science that deals with obtaining particles with nano dimensions (1–100 nm), addressing synthetic routes to obtain particles with changeable structures and sizes [79]. Thus, nanoscience is developed through nanotechnology and is working to discover new ways to combat challenges, especially those in the medical field. Therefore, the integration of nanotechnology in medicine has led to the emergence of nanomedicine and nano-delivery systems that allow for both the diagnosis of diseases and the administration of drugs in the right place. At this moment, nanomedicine-based drug delivery systems require advantages (Figure 4) and designs that allow for efficient drug delivery, favorable pharmacokinetics, high accuracy, and safety following administration [80,81,82,83]. Thanks to the unique properties of materials obtained by nanotechnology, they can revolutionize medical applications, as they can be used in diagnosing, treating, and delivering medical devices specific to different diseases. Thus, nanotechnology has led to a significant improvement in medical diagnosis by improving the sensitivity, accuracy, and speed of medical tests. For example, nanoparticles are commonly found in medical imaging (MRI, CT, PET) and improve the accuracy of diagnosis of various diseases. At the same time, nanoparticle-based biosensors can lead to early diagnosis of diseases. In the context of musculoskeletal disorders, nanotechnology may lead to faster diagnosis in the early stages of musculoskeletal diseases, due to the property of nanoparticles being functionalized with imaging agents, such as fluorescent dyes, or magnetic materials, thus obtaining high-quality images with much better contrast and real-time monitoring of disease progression and response to treatment. On the other hand, nanoparticles (NPs) developed by nanotechnology can also be used for efficient drug delivery. They have been observed to lead to improved drug pharmacokinetics, contributing to much better solubility and stabilization of active substances. NPs can also contribute to controlled drug delivery and targeting of specific cells, considerably reducing adverse effects. Thus, therapeutic efficacy is increased, the number of administrations is reduced, and the toxicity of the drugs decreases. Nanoparticles for drug delivery can be based on liposomes, proteins, polymers, and inorganic nanoparticles [84,85,86,87]. In the context of muscle disorders, including sarcopenia, nanomedicine offers opportunities to develop treatments with increased efficacy. Thus, DDSs, which have the ability to be transported at the cellular level, can be developed, as well as the surface-area-to-volume ratio of nanoparticles that may allow loading with growth factors, oligonucleotides, cytokines, or other agents to promote muscle tissue regeneration. Nanoparticles also allow the binding of structures to help targeted drug delivery [84,85]. Nanotechnology can also lead to scaffolds and nanofibers that lead to the repair of tissues, such as muscle fibers. In this sense, researchers have been able to develop functionalized scaffolds with myogenic cells and growth factors to restore muscle functions lost due to various diseases. These aspects will be developed in the following sections with a specific focus on sarcopenia [84,85,86,87].

However, there is a need for more studies on the use of nanomedicines, and not only for muscular disorders. Researchers need to assess the toxicity of nanomaterials to ensure that they do not endanger the health and life of patients, avoid bioaccumulation of the drugs at the sites of administration, overcome biological barriers and assess their mass production capacity, and achieve a low cost that allows their use in clinical trials and subsequent use by all categories of patients [84,85,86,87].

### 6.1. Nanoparticles and Liposomes

In terms of conventional drug delivery, both oral and injectable drugs are not yet sufficiently developed to achieve improved formulations; thus, the use of nanoparticles in medicine as therapeutic agents, delivery systems, and diagnostic systems is important and has become a necessity [88].

Nanoparticles can have different sizes (1–100 nm), structures (monolayer or multilayer), and shapes (spherical, tubular, hollow core, spiral, etc.). Also, depending on their chemical composition, they can be classified as organic, inorganic, carbon-based, or composite [89,90,91].

**Figure 5:**
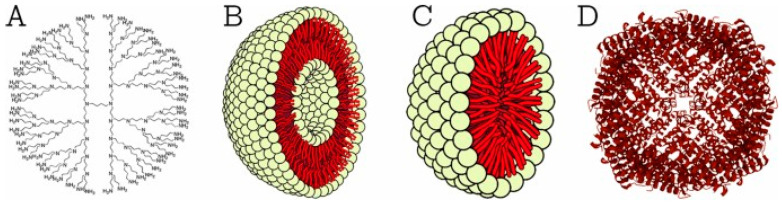
Classification of organic nanoparticles. (**A**) Dendrimers; (**B**) liposomes; (**C**) micelles; (**D**) ferritin. Reprinted from an open-access source [89].

Organic nanoparticles (Figure 5) are made of organic compounds such as lipids (liposomes), proteins (ferritin), and polymers (dendrimers, micelles), being approved by the body due to properties such as excellent biodegradability, non-toxicity, and high applicability in targeted drug delivery and cancer therapy. These particles are safe for use in biomedical applications and are naturally eliminated from the body, but they exhibit an increased sensitivity to external factors such as heat and light, making them unstable and unusable in certain environments [89,90,92].

Both liposomes and micelles are biodegradable and non-toxic organic compounds, and are sensitive to heat, light, and electromagnetic radiation. Both types of nanoparticles also have a hollow interior that allows them to be loaded with drugs [89,90].

Liposomes are spherical vesicles, synthesized by the hydration of dry phospholipids, which have an aqueous core surrounded by lipid layers, thus allowing the encapsulation of hydrophilic compounds in their core, as well as hydrophobic compounds in the lipid layer, and are used in the controlled administration of drugs. An important advantage of liposomes is their ability to fuse with the cell membrane, allowing them to release therapeutic content into the cytoplasm. Thus, liposomes can be used in the realization of intelligent delivery systems, offering also the possibility of targeted delivery of therapeutic agents (antioxidants, mtDNA, mitochondria-targeted molecules). Liposomes are classified as multilamellar, small unilamellar, and large unilamellar. The simplest liposome is formed by a lipid bilayer surrounding a hollow core, which allows the drug loading of the system. A multilamellar liposome consists of several lipid layers separated by aqueous spaces, while unilamellar vesicles have a single double layer surrounding the aqueous space. Also, liposomes allow the loading of multiple types of drugs, so that one therapeutic agent can be loaded into the lipid compartment while another can be inserted into the aqueous compartment [28,89,90]. Because of their structure, liposomes may have huge potential in treating sarcopenia. The double layer of phospholipids allows protection of the active substance once it enters the systemic circulation, ensuring gradual release. A preclinical study has shown that sphingomyelin-based liposomes exhibited an increased ability to ameliorate the symptoms of sarcopenia in accelerated-aging mouse models. The proliferation and metabolism of muscle cells were accelerated [93].

Inorganic nanoparticles are made of several types of materials, including metals, ceramics, and semiconductor materials. Metallic nanoparticles (e.g., silver, alumina, gold, copper, iron, zinc) can resist extreme conditions, making them usable in biomedical applications, including drug delivery and innovative therapies for various diseases. Metal oxides are more commonly used in drug delivery and exhibit advantageous properties over metal analogs. In this regard, specialists and researchers have made considerable progress in the use of these types of nanoparticles, observing the availability, biocompatibility, and stability of these types of materials. Metallic nanoparticles also allow therapeutic agents’ encapsulation and direct-to-target delivery, thus improving a drug’s effect against disease [89,90,94].

Carbon nanoparticles are composed exclusively of carbon (carbon, graphene, fullerene) and can have different shapes, such as nanotubes or spherical or ellipsoidal particles. In general, these materials are used to impart strength to other materials, thus forming composite materials [89,90].

Carbon nanotubes, ceramic nanoparticles, and metal nanoparticles have the potential to be used in drug delivery to treat sarcopenia. It has been shown that carbon nanotubes have the potential to be functionalized with a variety of therapeutic agents and to promote muscle tissue regeneration. As for metallic nanoparticles, they can be used in the targeted and controlled delivery of drugs and growth factors, promoting immunomodulation through macrophage polarization, leading to the amelioration or cure of sarcopenia [95,96,97].

### 6.2. Micelles and Dendrimers

Micelles are amphiphilic colloidal particles with a diameter between 5–100 nm, consisting of molecules with two regions having different affinities for water. The micelles are self-assembled and have both a polar (hydrophilic) and a non-polar (hydrophobic) part with a hollow core. In general, the polar part is inwardly oriented, while the non-polar part is outwardly oriented. They are structurally, kinetically, and thermodynamically stable due to the polymer chains present in the inner core and show an increased capacity to solubilize hydrophobic drugs. They are sensitive to pH and light; these properties are two key elements in the formation of drug delivery systems. These structures are used as drug delivery systems in the pharmaceutical industry, as they allow the delivery of lipophilic therapeutic agents in the inner core, while polar compounds can bind on the micellar surface. Micelles have been shown to have a high encapsulation capacity and improved solubility, and to help increase cell permeability; thus, an optimal concentration is delivered without drug resistance. One reason these types of particles are used in creating DDSs is that they are an easy and simple way to form ordered nanoscale structures that can deliver therapeutic agents in a controlled and targeted manner [89,90,98,99,100,101]. Recent studies have demonstrated the possibility of using micelles in muscle tissue regeneration. For example, polymeric micelles have been used to deliver anti-inflammatory drugs and anabolic agents, which have led to tissue regeneration. Another advantage of these particles in their use in the treatment of sarcopenia is their ability to modify the drug release profile. Micelles can thus be used in long-term therapies specific to degenerative diseases such as sarcopenia [102,103,104].

Dendrimers are a class of monodisperse, branched, polymeric macromolecules that have multiple chain-linked caplets, which allow specific chemical reactions to occur. The formation of a dendrimer starts from an initial atom (usually nitrogen) to which other elements (e.g., carbon) are bonded so that a spherical final shape is realized. The process is repetitive until successive layers are formed, and the size can thus be adjusted as needed. Thus, dendrimers have a three-dimensional structure that allows drug encapsulation and controlled release. Their structure plays an important role in their physical and chemical properties. Due to their large number of branches, dendrimers may have proven useful in the transportation of antibodies or hormones to their target site. Dendrimers have become widely used in clinical applications due to their structure, easily modified/conjugated surface, and decreased chance of cytotoxicity. They also have low bio-permeability and allow for controlled release of therapeutic compounds. Examples of polymers used in clinical trials in the form of dendrimers are poly(propyleneimine), polyethyleneimine, polyamidoamine, chitin, etc. [89,90,101,105].

Nanoparticles have numerous advantages in targeted and controlled drug delivery. Firstly, they can improve the bioavailability of drugs and provide sustained and controlled release, but they can also be used for targeted delivery of therapeutic agents. Such properties can be used and studied to create an optimal and effective treatment for sarcopenia that is well tolerated by patients and does not present adverse effects that worsen a patient’s health [28,88,89,90,94,98,99,100,101,105].

### 6.3. Targeted Delivery and Controlled Release

Targeted drug delivery is a new research idea in the pharmaceutical field which aims to deliver a therapeutic agent to a specific location without releasing it to other non-targeted areas. Thus, the purpose of a DDS is to keep the drug intact without causing any changes to the drug until it reaches the desired site and is released. Also, a DDS intended for targeted delivery must be stable, non-toxic, and biocompatible, and must not affect the drug’s pharmacological effects during release. Thus, targeted delivery systems are being studied, and it has been observed that they can improve bioavailability, pharmacokinetic parameters, and absorbability. Thus, researchers should consider that drug targeting must deliver a drug into the capillaries of the target tissue and to the target cells, including the organs and tissues for which the drug is intended. While this has many advantages, there are also barriers to overcome. For example, the drug may be eliminated too quickly from the body and must be taken much more frequently, with the risk of a high concentration of the drug accumulating at the target site. Also, manufacturing, storage, and administration should follow a well-established protocol so that the stability of the product is not affected [106].

Methods to streamline targeted drug delivery include passive, active, reverse, ligand-mediated, physical, and dual methods to enhance treatment effects and reduce side effects. These strategies use different approaches to ensure that drugs get to the right place in the body, at the right time, and with maximum effectiveness to more effectively treat complex conditions [106].

Controlled-release systems have been developed to make the administration of the pharmaceutical form better by reducing the dose and frequency of administration. Thus, the use of such systems is intended to reduce fluctuations in drug concentrations in blood plasma, reduce the adverse effects and toxicity of drugs, and increase patient comfort. Thus, to have an optimal therapeutic concentration, systems that deliver the therapeutic agent in a controlled manner are designed to have two parts: a loading dose and a maintenance dose. The initial dose, the loading dose, is intended to provide an immediate pharmacologic effect, while the maintenance dose is released slowly and steadily to maintain the effect. Thus, the latest delivery system generation has focused on finding innovative solutions, especially for water-insoluble drugs, developing long-term, patient-friendly technologies that do not jeopardize patient health when using nanoparticles and self-regulating drug delivery systems [21].

## 7. Polymer-Based Drug Delivery Systems

For the development of new drug delivery systems, polymers are a good option in the manufacture of such systems due to properties such as mechanical flexibility, availability, low density, degradability, and ease of fabrication, but also because they can be cost-effective compared to ceramic or metallic materials. Natural and synthetic polymers have gained attention for their pharmaceutical use due to unique properties such as biodegradability and bioabsorption capacity. Thus, bioabsorbable polymers such as hydrogels made from polylactic acid, polyglycolic acid, and their copolymers have been utilized in the manufacture of DDSs [107].

### 7.1. Biodegradable Polymers

A polymeric DDS has been defined as a formulation or device capable of introducing a therapeutic substance into the body, improving safety and control of the rate of release and targeting the site for maximized local effect. A classification of the polymers used in the manufacture of DDSs is presented in Table 3 [107].

Biodegradable polymers, either synthetic or natural, can bring a considerable breakthrough in the manufacture of DDSs. As for sarcopenia, the polymers were made to obtain scaffolds. Therefore, biodegradable polymers have proven to have a high capacity for controlled drug release so that an optimal drug concentration can be maintained in the bloodstream without causing adverse effects and without the need for frequent dosing of the drug. Biodegradable polymers also do not produce toxic compounds upon degradation and, at the same time, protect the drugs from contact with the aggressive environment of the human body. Some biodegradable polymers can be conjugated for use in targeted drug delivery, thereby reducing the risk of side effects and increasing therapeutic efficacy. However, more studies need to be conducted in order to mitigate some of the problems that polymeric DDSs may cause in the context of sarcopenia treatment. Control of the degradation rate is important but is difficult to control; thus, the drug may be affected. Also, the polymer used in making the DDS must be chosen so that it can be used with a particular type of drug [107,108,113,119,121,124,125]. For muscle regeneration, polymers are manufactured as scaffolds that must offer the following characteristics. Biocompatibility is a must, and the mechanical properties of polymers should resist cell microenvironment stress. Also, materials used in scaffold manufacturing must promote cell adhesion and cell growth [126].

### 7.2. Hydrogels and Smart Polymers

Hydrogels are made of hydrophilic polymers composed of three-dimensional networks, which have the property of retaining water and swelling in physiological environments without dissolving, maintaining the structure by chemical or physical cross-linking mechanisms. Hydrogels can be used in the manufacture of DDS as they exhibit physical and chemical properties that allow them to be used in such applications. They are also similar to the extracellular matrix (ECM) structure and enable their use in biomedical applications (drug delivery, tissue engineering, wound healing, etc.). Thus, hydrogels have proven to have good, controlled dissolution capacity, sustainably releasing drugs (small molecules, macromolecules, or cells), protecting them from the aggressive environment of the body, and offering the possibility of solving formulation problems. Hydrogels can be used in various forms, including as dressings, regenerative medicine, and hygiene products.

In drug delivery, hydrogels face problems related to biocompatibility, safety, and release of active substances. Although natural and synthetic polymers exist to combat some of these negative aspects, control over drug release in physiological environments remains challenging, along with mechanical stability, biodegradability, and targeted delivery [127,128,129,130].

In this way, researchers need to optimize drug delivery mechanisms using hydrogels so that the therapeutic effects are superior and to overcome the challenges mentioned above. Parameters such as the structure and mesh size of hydrogels influence drug release. The eyes in the polymeric fiber network allow the diffusion of liquids, solvents, and drugs. Rapid diffusion occurs when the mesh size is large, whereas if the size of the drug molecules is similar to the mesh size, diffusion is significantly slowed down. If the molecules are much larger, the drug is captured, and the release is achieved by mesh modification through lattice degradation, controlled swelling, or application of mechanical deformation. By controlled degradation of the mesh, the mesh size can increase and thus allow the drug to be released. Degradation methods can be achieved by hydrolysis or enzymatic activity triggered by external stimuli such as UV light or magnetic fields. Controlled swelling of the hydrogel is another way to release drugs. The size of the eye enlarges and allows diffusion of the active substance through the network. Hydrogel swelling can be influenced by parameters such as pH or temperature. Mechanical deformation can temporarily increase the mesh size and allow for drug release. This method may involve the application of direct stresses or deformation using ultrasound or magnetic fields [131].

The ability of these materials to interact with external factors, such as chemical, physical, or biological factors, has led to further research and the emergence of “smart” polymers. Smart polymers have the ability to change their properties when exposed to different stimuli, such as chemical stimuli (e.g., pH, redox stimuli, redox), physical stimuli (e.g., temperature, electric stimuli, photo-responsive stimuli), and biological stimuli. Polymers that react to multiple stimuli simultaneously are called dual-stimulus polymers [132,133].

pH-responsive polymers undergo structural and property changes as a function of the pH of their environment due to the presence of functional groups in their structure that lead to inorganization or deionization depending on pH changes. Thus, pH-influenced polymers can be classified into acidic or basic polymers, which are the types of polymers that can be used in biomedical applications. Ionic strength can influence the swelling and dehydration of polymers or help self-assembly into complex structures depending on the interaction of the functional groups in the polymer chains. Redox stimuli cause polymers to respond to changes in the redox potential in the surrounding environment. Thus, redox-active functional groups in the polymer chain may undergo oxidation or reduction processes. This phenomenon can influence the solubility of polymers, which is an important aspect of controlled drug delivery. pH-sensitive hydrogels have been used to regenerate muscle function. Thus, carboxymethyl chitosan, oxidized chondroitin sulfate, and cystamine dihydrochloride were cross-linked to obtain an injectable hydrogel that delivers stem cells together with extracellular vesicles (EVs). In the presence of pH or ultrasound, the hydrogel released EVs in a controlled manner and thus greatly improved muscle function and mass in vivo [134].

Because muscles are sensitive to electrical stimuli, researchers worked to obtain an aerogel that stimulates the growth and differentiation of myoblasts. Muscle fiber size increased and muscle strength and function were regained experimentally in vivo, which could represent a potential treatment for sarcopenia [135].

Electro-responsive polymers change their properties due to electrical signals, while photo-responsive polymers change their properties in the presence of light.

To restore muscle function, researchers have also been working on a magnetic hydrogel as a potential treatment for sarcopenia. This hydrogel is designed to be injected directly into the muscles with a wearable device to stimulate the muscles. It has been observed that following the use of such a device and following constant stimulation, muscle function and muscle mass were significantly improved [136].

## 8. Peptide- and Protein-Based Delivery Systems

Small molecules, proteins, and oligonucleotides have attracted researchers’ attention to the development of DDSs. Thus, drug delivery systems based on peptides and proteins have emerged.

Peptide-based systems have been shown to have many advantages over other types of DDSs. Excellent biocompatibility, lack of toxicity, and easily controllable physical and chemical properties make peptide-based DDSs (PDDSs) ideal candidates for such applications. A major advantage is the ability of certain types of peptides to penetrate cells without membrane denaturation, making them effective and safe DDSs. These peptides are called cell-penetrating peptides (CPPs) and are used as effective and safe delivery systems. CPPs are taken up by cells through two phenomena called endocytosis and direct translocation. With the help of peptides, DDSs can be designed to target a target tissue and to be sensitive to stimuli. Targeted delivery of PDDSs has the ability to reduce adverse effects and may improve therapeutic efficacy. Also, the fact that peptides can be sensitive to different stimuli (chemical, physical, biological) may enhance control and understanding of modifications that may lead to the development of more evolved delivery systems. Antisense nucleotide peptides have been investigated for delivery to skeletal muscle to treat muscle disorders such as sarcopenia and have shown therapeutic effects in mouse models. Likewise, the prequel Ghrelin polypeptides have been used for the delivery of growth factors, as well as to control IGF-1 expression to maintain muscle mass. This polypeptide, however, has a short half-time in the body and cannot be used for sustained hormone delivery [137,138].

In order to be usable, PDDSs require further studies focusing on the stability of the systems, improved pharmacokinetics, and their behavior in the body. It has been observed that cell membrane penetration has led to increased levels of amino acids or peptides in cells and has caused renal problems and the production of proteins with increased toxic potential. Thus, in order to be used safely, these challenges need to be studied by researchers to ensure effective treatments that do not cause severe adverse effects for patients [139,140].

Fibrillar proteins (e.g., collagen, keratin, elastin, etc.) have lately been studied for use in drug delivery. These materials have similar properties and can be processed in different forms, such as nanoparticles, nanofibers, microbeads, sponges, scaffolds, or films. The delivery of drugs via proteins requires careful attention in order to have effective delivery and optimal therapeutic effects. Thus, there is a need to ensure that the drug is maintained and released in a controlled manner over a long period of time and at optimal concentration. Collagen was used in the form of nanoparticles placed in a scaffold to improve muscle contractile function, while fibrin- and collagen-based composite gels were used to deliver myoblasts, which formed myotubes resembling natural muscle fascia. The ability of collagen, fibrin, and keratin to promote the growth and differentiation of muscle cells was also studied, and they were shown to promote muscle tissue repair. These proteins have also been used for gene delivery, as well as cell delivery, to achieve therapeutic effects in damaged muscle [111,141,142].

The first limitation of these types of DDSs is the proteolysis and renal elimination of drugs, so they require modifications to protect them from physiological conditions. Proteins resemble peptides, being derived from similar building blocks. Also, polymers made from proteins have proven to have much better biocompatibility than synthetic polymers, to have high biodegradability, to not cause toxicity, and to have low antigenicity. However, they show instability, low mechanical properties, and have irreproducible properties [143,144].

Also, it was observed that peptide and protein drug delivery systems are unstable once they enter the systemic circulatory system, so these DDSs must avoid enzymatic degradation, opsonization, and the reticuloendothelial system (RES). Also, non-selective protein accumulation, maintenance of solubility, activity, and targeting of the site of delivery, cell uptake, and release are still being researched to be enhanced. Because of these challenges, these DDSs are delivered through transdermal and injectable routes. The gastrointestinal tract is not an option because it may promote a quick degradation of a DDS and a lower efficacity. In order to overcome some of the limitations and to develop high-performing DDSs, peptides, and proteins as drug carriers can undergo modifications to enable their use in such a context. Modifications can be chemical (amino acid modification and hydrophobization), improvement of cell membrane penetration, and incorporation into DDS-type matrices (emulsification, extrusion, spray drying, and polymerization) [145].

## 9. Emerging Delivery Technologies

### 9.1. Gene Therapy and Nucleic Acid Delivery

Due to the need for new and effective therapeutic methods, gene therapy has been developed for the treatment of many diseases. In comparison with classical treatment methods, gene therapy makes it possible to correct genes so that the effect on diseases is sustained. Gene therapy consists of using exogenous nucleic acids to modify disease-associated genes via different processes such as deleting, replacing, suppressing, and knocking genes [146].

In recent years, nucleic acids (NAs) have gained attention as an efficient strategy to treat diseases with clinical use, namely vaccination. For a vaccine example, Bing Fu et al.’s study focused on improving muscle mass in *Epinephelus coioides* using a myostatin autologous nucleic acid vaccine and found that it promoted muscle growth [147]. It has been observed that NAs have the potential to be used in gene therapies to treat various diseases. Thus, NAs can act directly or indirectly on cellular functions and be designed to treat any disease at any stage. At the same time, NAs can use cellular processes or encode information (e.g., messenger RNA can encode recombinant proteins) to correct cell misbehavior. One challenge in this regard is the susceptibility of NAs to degradation due to the environment. NAs are negatively charged and vary in size, making passive diffusion into cell membranes difficult, therefore requiring delivery vectors to transport them to the target. Thus, a DDS designed for their delivery must help NAs to penetrate cells through lipid bilayers and provide protection against premature degradation. At the same time, the immune system is another obstacle to NA delivery. It associates NAs with pathogens and thus triggers an immune response to neutralize them. Nucleases and degrading enzymes intervene and break down foreign NAs, and this represents a major challenge in their efficient delivery [146,148].

Nanomaterial-based technologies for NA delivery must ensure the success of gene therapy by overcoming cellular and subcellular barriers to deliver the therapeutic payload to the site of action (e.g., cytoplasm, mitochondria, nucleus). In addition to overcoming biological barriers, researchers focused on increasing the retention time of nanoparticles in systemic circulation and reducing immune clearance. Using nanoparticles, the aim is to deliver NAs such as messenger RNA (mRNA), small interfering RNA, micro RNA (miRNA), plasmid DNA, and antisense oligonucleotides (ASOs) by enhancing the nanoparticle surface with targeting ligands that bind to receptors on target cells. In cells, mRNA can induce protein expression, while siRNA and ASO inhibit gene expression. Both RNA and DNA can be used to manipulate gene expression, and a concrete example is in the targeting of mitochondria to correct mitochondrial genetic defects [148,149,150,151].

In this regard, nanoparticles for NA delivery include lipid nanoparticles (LNPs), polyionic complex micelles (PICs), pH-sensitive polymeric nanoparticles, and biodegradable polymeric nanoparticles. LNPs are composed of ionizable cationic lipids that surround NAs and protect them from degradation, although their delivery efficiency is low and they pose a major risk of cytotoxicity. PICs rely on electrostatic attraction for NA encapsulation, being versatile and allowing adaptation to environmental changes (pH or redox conditions). However, PICs have a high degradation rate, and they require improvements to be more efficient in their use as delivery systems. pH-sensitive nanoparticles have been designed to release NAs depending on pH changes. Thus, decreases in cell pH can lead to the release of therapeutic cargo. Regarding the use of biodegradable polymers, PLGA has been studied in this regard. Thus, the ability to protect NAs and to gradually release them following polymer degradation has been demonstrated, but chemical modification of PLGA to obtain optimal properties is difficult, and degradation products can cause inflammation. In addition to the use of nanocarriers, improvement strategies also target nucleic acid modifications. Thus, chemical modification can be carried out at nucleotide bases and at the sugar–phosphate skeleton level to make NAs more stable and decrease the body’s immune reaction [149,150].

In muscle disorders, gene therapy uses approaches such as exon skipping and the CRISPR/Cas9 system. Exon skipping uses antisense oligonucleotides (ASOs) to specifically recognize and link certain mRNA sequences, blocking protein maturation or altering pre-mRNA splicing. ASOs show potentially promising features, but limitations have also been identified, such as instability, poor distribution, and difficult cellular internalization. To overcome these problems, the chemical structure of ASOs was modified, thereby increasing their stability. Polymers such as PEG (polyethylene glycol), PEI (polyethylenimine), PMMA (poly(methyl methacrylate)), and PLGA (poly(lactic-co-glycolic acid)) were tested in vitro as nanosystems for gene therapy [152].

Also, Philippou et al. [153] demonstrated that RNA aptamers can be used to obtain gene therapy targeting muscle cells. Since gene therapy using oligonucleotides is effective, but selective delivery of the treatment to muscle has been a major challenge, decreasing the effectiveness of the treatment, the researchers used a technique called SELEX (Systematic Evolution of Ligands by Exponential Enrichment) to identify a skeletal muscle-specific RNA aptamer. The SELEX technique contributed to the identification of the RNA aptamer A01B, which demonstrated a high affinity for skeletal muscle cells. This was demonstrated by in vitro tests performed on murine skeletal muscle cells (C2C12), in which it is well internalized, and on liver and kidney cells, where the affinity was low. A01B was able to concentrate in the cytoplasm of skeletal muscle cells, and this highlights that it is highly specific and can be used as a target for muscle therapy. In experimental tests and in vivo mouse models, the aptamer retained its functionality, efficiently entering skeletal muscle after local administration, with clear specificity for skeletal muscle compared to negative control. Thus, the A01B aptamer may represent a key factor in the development of more effective targeted therapies for affected skeletal muscle, minimizing adverse effects due to its high specificity.

Given the targeted treatment of sarcopenia, a cis meta-analysis [154] and a randomized Mendelian study [155] led to some key observations in this regard. The meta-analysis focused on studying the effects of plasma proteins on the risk of developing sarcopenia in women. Thus, five proteins that are associated with sarcopenia were identified, but only two (TNF12 and HGF) have been used for targeted therapy in clinical trials. On the other hand, Mendelian Randomization Analysis revealed five other plasma proteins—leukocyte immunoglobulin-like receptor subfamily B subfamily member 2 (LILRB2), asporin (ASPN), contactin-2 (CNTN2), ecto-ADP-ribosyltransferase 4 (ART4), and superoxide dismutase 2 (SOD2)—that may be associated with sarcopenia and could represent new targets in the manufacture of drugs for this disorder.

In the context of treating sarcopenia, there are no approved treatments, and current interventions (e.g., exercise or supplements) have significant limitations. In this regard, a recent study by Yu et al. used tetrahedral framework nucleic acids (tFNAs) to ameliorate or cure sarcopenia [156]. Thus, tFNAs are a type of DNA-based nanomaterials that have demonstrated an enhanced ability to ameliorate apoptosis in cells and tissue. A significant improvement in muscle function was observed during in vitro and in vivo testing, which also revealed morphological changes in muscle fibers. Also, a study by Yanai et al. collected data on the importance of miRNA presence in the stages of sarcopenia [157]. Thus, the research showed that the expression of miRNA could influence the development of sarcopenia, which should be considered for future studies so that miRNA can be used to treat sarcopenia. In another study, researchers pointed out that non-coding RNAs are involved in the evolution of sarcopenia by regulating TGF-β/BMP, IGF-1, and MRF signaling pathways, and non-coding RNAs may represent biomarkers and therapeutic targets for this condition [158]. However, further studies are needed to develop treatments with the potential to ameliorate or cure sarcopenia.

### 9.2. Cell-Based Delivery Systems

Cell-based therapy is a recently developed method with huge potential for treating many conditions, involving the use of cells as delivery agents. Cells that can be used in this type of therapy are red blood cells/erythrocytes, platelets, leukocytes, lymphocytes, monocytes, neutrophils, macrophages, and stem cells. An example of an FDA-approved treatment is the transfer of genetically reprogrammed adoptive T cells to fight lymphoid cancers. In the fight against sarcopenia, stem cells are used to regain function and muscle mass affected by this disease. Thus, it was proposed that seven satellite cells be transplanted together with a myofiber, obtaining more than 100 new myofibers, which regenerate muscle tissue. Another study focused on mitochondrial transplantation to improve skeletal muscle health and implicitly for the treatment of sarcopenia [159,160].

An advantage of this type of therapy is the ability of the cells to naturally migrate and localize to specific target tissues, providing a more uniform biodistribution and targeted delivery compared to other developed methods. However, cell therapy also faces translational issues just as with gene therapy, including safety concerns and high production costs. Another problem is the cell source. Thus, appropriate sources (autologous, allogeneic, or xenogeneic) of cells must be identified to obtain a viable and safe product. Products must also be sufficiently viable to ensure the required therapeutic profile. Genome editing, synthetic biology, and using biomaterials to create therapeutic strategies with superior, effective, and safe properties are crucial in cell therapy development. Other challenges are the processes of in vitro cell isolation and loading of therapeutic agents [161,162,163].

### 9.3. 3D Printing and Customized Drug Delivery

Three-dimensional printing is a technology that has developed more and more in the manufacturing industry as it offers numerous advantages, such as increased productivity and efficiency, but it also considerably reduces production costs and human error. Thus, it has also proved useful in the biomedical industry, being used in the production of medical devices with both therapeutic and diagnostic roles. In terms of DDS development, 3D printing has led to pharmaceutical dosage forms, which have required a short manufacturing time and few resources. DDSs manufactured by 3D printing were found to have dosing flexibility and exhibited increased bioavailability, and it was noted that they can be used to create customized formulations tailored to individual patients’ needs. This technique could also be used in the formation of complex formulations for therapies, which featured multiple therapeutic agents that sustained long-term therapy [164,165]. Three-dimensional printing can also be used to obtain transdermal therapeutic systems, vaginal and rectal delivery systems, implants, scaffolds, microceuticals, capsules, films, and hydrogels, as well as tissue and organ printing [166,167].

Both microchips and 3D-printed scaffolds can be used to create implantable drug delivery systems, which can also be used in cell delivery or gene therapy. For example, 3D scaffolds, which can be made of nanofibers or porous matrices that respond to stimuli such as temperature, can promote cell adhesion and allow cells to proliferate, differentiate, and migrate, which is an advantage in creating a system to treat sarcopenia. Thermosensitive polymers can also lead to the controlled release of drugs with low water solubility, with the scaffold improving this property of the drug and increasing its bioavailability. These scaffolds can be used in local drug delivery by implantation at the desired site, releasing the active substance in a controlled manner. Microchips can also be used in the local administration of different therapeutic formulations. This can be achieved by the membrane covering the drug-loaded reservoir, which dissolves over time. Microchips can also be used in both drug delivery and microfluidic cell therapy, which can be advantageous for muscle regeneration [168,169].

Many studies have focused on obtaining scaffolds that mimic muscle structure. On the other hand, the hydrogels and polymers used to make 3D-printed scaffolds can be filled with cells, such as myocytes, which also promote muscle function and mass. Three-dimensional structures have also allowed for the organization and emergence of contractile units (actin and myosin filaments), leading to muscle myotubes for muscle tissue repair [148,149].

## 10. Preclinical and Clinical Studies

### 10.1. Key Findings from Animal Studies

Sarcopenia is a degenerative condition and a worldwide public health problem, occurring especially with aging. Currently, sarcopenia is kept under control through physical therapy and supplementation, as well as radical lifestyle changes, but these strategies are not approved for specific use in treating the condition. As research into sarcopenia and its mode of onset as a condition in the elderly continues to increase due to the knowledge of complex molecular mechanisms, new therapeutic targets have begun to be studied to realize new drug formulations and diversification of treatment approaches [170].

At the moment, therapeutic agents have been evaluated in preclinical studies for their therapeutic potential. Thus, exekines (interleukin 6, interleukin 15, fibroblast growth factor, TNF-α, irisin, etc.) have started to be evaluated as potential therapeutic agents in sarcopenia. Thus, exekines were observed to have autocrine, paracrine, and endocrine effects [171].

On the other hand, selective modulators for androgen receptors, testosterone, and myostatin inhibitors have been shown to provide an increase in muscle mass [171]. Regarding testosterone treatment, the results of studies have varied depending on different parameters such as patient age, pre-treatment testosterone levels, and methods of administration. This treatment approach does not come without increased risks of complications, such as gynecomastia, sleep apnea, edema, and prostate cancer [172,173]. Branched-chain amino acids (e.g., valine, leucine, isoleucine) may decrease the risk of muscle atrophy in sarcopenia. They stimulate protein synthesis and thus inhibit protein degradation. Leucine-based supplements have been shown to be well tolerated and lead to significant improvements in elderly patients [174,175,176]. Although they are effective, a way must be found to integrate them into a complex therapeutic formulation. Natural compounds extracted from plants, such as curcumin, uroylitin A, ursolic acid, vitamin D, tomatidine, and green tea catechins, show therapeutic potential for sarcopenia, but more studies are needed to concretize the beneficial effects [170,171,173,177,178,179,180,181].

### 10.2. Clinical Trials and Human Studies

#### 10.2.1. Myostatin-Targeted Delivery Systems

Myostatin (MSTN) is a growth differentiation factor-8 (GDF-8) and functions as a negative regulator of muscle mass by binding to receptors on muscle fiber membranes. Thus, inhibition of MSTN represents a strategy to stimulate muscle growth and prevent muscle wasting, as in sarcopenia. In a phase II study, Landogrozumab, a monoclonal antibody targeting MSTN, demonstrated a potential to increase muscle mass and partially improve muscle function and mobility in patients over 75. During the study, patients were injected with 350 mg of Landogrozumab every 4 weeks for 20 weeks, followed by 16 weeks of observation. Other studies focusing on other MSTN inhibitors have demonstrated good safety profiles in preclinical studies but have failed in clinical trials due to a lack of efficacy in terms of increasing muscle mass or muscle function [78,182].

#### 10.2.2. Activin Receptor Antagonists

Activin receptor (ACVR2) antagonists have succeeded in blocking the interaction between MSTN and ACVR2, thus preventing the development of MSTN-induced muscle atrophy. The Novartis Institutes for BioMedical Research developed a monoclonal antibody called bimagrumab to block a single-receptor pathway, which led to only a small increase in muscle mass. In the phase II clinical trial, patients with sarcopenia received 30 mg/kg of bimagrumab for 16 weeks, which increased muscle mass, and a physical grip test showed improved muscle function [78,183,184,185]. Further studies were carried out to concretize the results. Thus, bimagrumab led to a considerable increase in muscle mass [185,186,187].

#### 10.2.3. Follistatin Fusion Proteins and Gene Therapy

Follistatin (TGFp) has at least six isoform forms, obtained by alternative gene splicing and post-translational proteolysis, and has the ability to bind to heparin binding sites (HBS). Acceleron Pharma designed an ACE-083 fusion protein with IgG Fc fused to FST291, and in phase I clinical trials, the treatment significantly promoted targeted muscle growth, but not muscle strength.

Intramuscular gene transfer of follistatin fusion proteins promoted muscle hypertrophy. FST-344 gene therapy was administered using the AAV1 viral vector, developed by Nationwide Children’s Hospital and Milo Biotechnology, and was tested in clinical trials in patients with muscular dystrophy, with significant improvement in muscle function and no adverse effects [78,188].

#### 10.2.4. ACE Inhibitors

ACE inhibitors have been shown to decrease the chances of developing sarcopenia by blocking the production of AngII. ACE inhibitors are generally used to treat cardiovascular disease and prevent strokes. By promoting blood supply to muscle cells, ACE inhibitors decrease inflammation and increase IGF-I levels, leading to an increase in the number of mitochondria. In current studies, ACE inhibitors significantly improved muscle mass and function in 70-year-old patients with sarcopenia [78,189,190,191,192].

Although studies are not yet completed, preliminary data are still insufficient to demonstrate the ability of the designed drugs to treat sarcopenia. Thus, studies should be carried out to highlight potential adverse effects, and other studies should be conducted to lead to an improved and more effective effect in developing muscle mass and strength in patients diagnosed with sarcopenia [78].

## 11. Regulatory and Ethical Considerations

The development of drug delivery systems through nanomedicine has become a topic of intense academic research, directly impacting the public health system and human health. A first generation of pharmaceuticals based on nanomaterials has already been successfully introduced on the drug market, contributing to achieving a higher standard of living for patients. Although drugs of this type exist on the market today, the FDA and EMA have not regulated the requirements for preclinical and clinical testing of delivery systems. Thus, the development of a DDS begins with preclinical testing, followed by the filing of an Investigational New Drug (IND) application so that clinical trials can be initiated. Understanding the manufacturing process and detailed product characterization is necessary to successfully commercialize DDSs. It should also be recognized that nanotechnology has found its application in the medical field, and currently, authorities and experts in the field are working on solutions and new implementations of procedures to adapt to this new series of newly designed and developed drugs and to allow for their commercialization [193].

## 12. Conclusions

Being a degenerative disorder, sarcopenia is characterized by progressive loss of muscle mass and strength with increasing age and is associated with numerous complications such as fractures, disability, and a decreased quality of life. However, there is no clear treatment regimen for this condition, and dietary supplements, a balanced diet, and exercise are not sufficiently effective in halting the disease. This review highlights controlled release systems (DDSs) that may represent an advanced and personalized strategy in drug delivery for sarcopenia to provide advantages such as efficacy, ensuring precise and regulated release, and targeted delivery. However, for the development of DDSs capable of being used in sarcopenia therapy, first, the molecular mechanisms of the disease need to be understood, and then aspects such as route of administration, biomolecule interactions, interaction with body fluids, and other aspects related to loading of delivery systems and precise targeting of muscle tissue need to be taken into account. At this time, the results of clinical studies have demonstrated efficacy in restoring muscle mass and function, but the possible adverse effects that may occur after treatment must also be considered [194].

## Figures and Tables

**Table 1:** Factors influencing the choice of administration route. Created based on information from [48].

Principles	Factors
Patient-related principles	- Age - Sex - Body mass index (BMI) - Presence of comorbidities or complications - Level of disease awareness - Experience in the past
Medication-related principles	- Injection site - Dosage, frequency, and administration time - Formulation characteristics
Pharmacoeconomic-related principles	- Socioeconomic status of the patient - Healthcare staff and human resources

**Table 2:** Advantages and disadvantages of transdermal drug delivery systems. Created based on information from [64].

Advantages	Compliance and easy use for patients Higher effectiveness because TDDs avoid first-pass metabolism and the aggressive environment of the gastrointestinal tract Longer release time for the therapeutic agent TDDs are non-invasive TDDs have fewer side effects The dosage is known and established
Disadvantages	Skin could get injured or hypersensitive The concentration of drugs is lower compared with other DDSs TDDs are not recommended for high-dosage therapeutic agents Bigger molecules are harder to absorb, and ionic active substances cannot be delivered by this route

**Table 3:** Classification of polymers used in DDS manufacturing.

Classification	Polymer	Characteristics	Refs.	
Natural	Chitosan	It has functional groups in its structure (amino, C, OH) that enhance the solubility of the material, its functionalization, and its interaction with the medium. It is a bioadhesive, bioresorbable, biodegradable, and biocompatible material. It is a promising material for manufacturing nanoparticles, hydrogels, and membranes because of its higher capacity for drug encapsulation. It is used to deliver small molecules or polyanionic molecules like nucleic acids of DNA and RNA. It has higher hydrophilicity, a higher degree of swelling, poor solubility, low thermal stability, and lower ductility, which represents some of its limitations. Nanoparticles can be loaded with anti-inflammatory substances that are released in a controlled manner in the muscles. A recent study showed that the release of curcumin aldehyde via chitosan nanoparticles improved muscle atrophy. It also showed an increased cell attachment capacity and was also shown to deliver oligonucleotides to relieve the symptoms of muscle disorders. Because of its high conductivity, it can be used to manufacture artificial muscles.	[107,108,109,110,111]	
	Alginate	It is a biocompatible, biodegradable polymer with low toxicity. It can be easily manufactured. It is also used as a stabilizing agent in drug delivery systems. It is soluble in neutral and alkaline conditions. At acid pH, the release of drugs can be inhibited. Increases bioavailability of drugs. Can be manufactured as gels, matrices, membranes, and micro- and nanospheres. Can be used for protein delivery, cell encapsulation, and various low- and high-molecular-weight drugs. Alginate-based composite materials have been used in numerous clinical studies for the fabrication of 3D-printed muscle fibers to regenerate muscle tissue by delivering myoblasts.	[107,111,112]	
	Cellulose	It is a biodegradable, biocompatible polymer. It has high mechanical and thermal stability. It has a rather low solubility, which is a limitation that disadvantages it in biomedical and pharmaceutical applications. It can be used as an excipient in drugs to control the rate of release and optimize the concentration of the drug delivered. Derivatives with improved properties can be obtained. Nanocellulose is a derivative that shows superior properties; it is non-toxic and biocompatible and favors electrostatic adsorption due to OH groups and negative charges at the inter0P-5face. It can be absorbed on the drug or biomolecule surfaces. It is cost-effective. Composite materials based on cellulose and carbon nanotubes have been used to fabricate scaffolds for muscle tissue regeneration.	[107,113,114]	
	Hyaluronic acid	It is a natural, biocompatible polymer found in the extracellular matrix. It is used in the pharmaceutical field due to its anti-inflammatory, tissue repair and regeneration, and anti-aging potential. It has high biocompatibility and is non-toxic and biodegradable. It enhances drug solubility and pharmacokinetic parameters and improves sustained release and drug targeting. A hyaluronic acid-based delivery system can come in many pharmaceutical formulations, such as nanoparticles, nano-emulsions, criogels, and microneedle patches. It was used in in vivo tests for pentamidine delivery into muscle cells. Thus, it was demonstrated that the nanoparticles were internalized with low toxicity, successfully delivering pentamidine. Also, in another study, it was used to deliver the peptide sequence bsp-RGD(15), a high molecular weight heparin for growth factor sequestration, and a cross-linker that can be dissolved by matrix metalloproteinase to enable cell-dependent remodeling. This study showed a large regeneration of muscle mass and vascularization in mouse models.	[107,115,116]	
Synthetic	Polylactic acid	It is easily obtained from wheat, rice, and corn. It is biocompatible, biodegradable, and non-toxic. The intermediate, called lactic acid, can be easily metabolized by the body. Due to its excellent biodegradability, it can be used as a DDS. Its use in DDSs can lead to a controlled release of an optimal drug concentration and reduce the adverse effects of the drug. It has also been manufactured for cell culture and use in cell-based gene therapy. Together with its derivatives, it was tested in vitro for muscle regeneration. Thus, nanofibrous sheets with progenitor myoblasts administered to mice were made.	[66,117,118]	
	Poly (lactic-co-glycolic acid)	It is a hydrophobic, biodegradable, non-toxic, and biocompatible copolymer obtained by the amalgamation of polylactic acid (PLA) and polyglycolic acid (PGA). It has improved solubility compared to PLA and PGA. It can be manufactured in several shapes and sizes. It is used to encapsulate a wide range of drugs and molecules. An in vivo study conducted by Hyun Joo Jang et. al studied the effect of camphor on muscle atrophy. In doing so, the researchers observed that it relieved some of the symptoms. For efficient delivery, they used PLGA microspheres loaded with camphor, which they injected intramuscularly into mouse models, observing that the microspheres were able to significantly regulate skeletal muscle atrophy in vivo.	[119,120]	
	Polycaprolactone	It has low biodegradability in the physiological environment. Can be used as nanoparticles, films, microparticles, and fibers. It can be used as an implantable DDS and scaffold. It has low solubility in water. Scaffolds have been made to mimic the alignment of cells to mimic natural tissue but also to promote cell proliferation and differentiation, forming myotubes.	[107,121,122,123]	
